# Scaling production of GFP1-10 detector protein in *E. coli* for secretion screening by split GFP assay

**DOI:** 10.1186/s12934-021-01672-6

**Published:** 2021-09-30

**Authors:** Carolin Müller, Chika L. Igwe, Wolfgang Wiechert, Marco Oldiges

**Affiliations:** 1 Institute of Bio- and Geosciences, IBG-1: Biotechnology, Forschungszentrum Jülich GmbH, Jülich, Germany; 2 Institute of Biotechnology, RWTH Aachen University, Aachen, Germany; 3 Computational Systems Biotechnology (AVT.CSB), RWTH Aachen University, Aachen, Germany

**Keywords:** *Escherichia coli*, Inclusion body, Fed-batch, Split GFP assay, *Corynebacterium glutamicum*, Signal peptide screening, Cutinase

## Abstract

**Background:**

The split GFP assay is a well-known technology for activity-independent screening of target proteins. A superfolder GFP is split into two non-fluorescent parts, GFP11 which is fused to the target protein and GFP1-10. In the presence of both, GFP1-10 and the GFP11-tag are self-assembled and a functional chromophore is formed. However, it relies on the availability and quality of GFP1-10 detector protein to develop fluorescence by assembly with the GFP11-tag connected to the target protein. GFP1-10 detector protein is often produced in small scale shake flask cultivation and purified from inclusion bodies.

**Results:**

The production of GFP1-10 in inclusion bodies and purification was comprehensively studied based on *Escherichia coli* as host. Cultivation in complex and defined medium as well as different feed strategies were tested in laboratory-scale bioreactor cultivation and a standardized process was developed providing high quantity of GFP1-10 detector protein with suitable quality. Split GFP assay was standardized to obtain robust and reliable assay results from cutinase secretion strains of *Corynebacterium glutamicum* with *Bacillus subtilis* Sec signal peptides NprE and Pel. Influencing factors from environmental conditions, such as pH and temperature were thoroughly investigated.

**Conclusions:**

GFP1-10 detector protein production could be successfully scaled from shake flask to laboratory scale bioreactor. A single run yielded sufficient material for up to 385 96-well plate screening runs. The application study with cutinase secretory strains showed very high correlation between measured cutinase activity to split GFP fluorescence signal proofing applicability for larger screening studies.

## Background

The split GFP assay is a versatile tool for protein detection. In contrast to full length reporter proteins, only the 11th $\beta$-sheet of a superfolder GFP is used as a tag for detection. Since the $\beta$-sheet consists of only 16 amino acids connected to the target protein by a small peptide linker, the impact of the tag on solubility and folding of the target protein is minimized [1]. The other part of the superfolder GFP, GFP1-10, is non-fluorescent itself because the GFP chromophore is not formed without residue E222 [2], which is located in the GFP11-tag. Only in the presence of an accessible GFP11-tag containing this residue, GFP1-10 and GFP11 are self-assembled and fluorescence can be measured after chromophore maturation [1]. For *in vitro* protein detection, GFP1-10 can be produced separately in *Escherichia coli* as inclusion bodies. After cell disruption the inclusion body fraction is purified, GFP1-10 is refolded and ready for application [3, 4].

In recent studies, the system was optimized for faster fluorescence formation by prematuration of the GFP1-10 [5, 6]. This could drastically reduce incubation times but requires additional purification steps. The split GFP assay can also be used *in vivo* e.g. for solubility assay [3] and was extended to other fluorescence proteins [7, 8]. A broad review of recent developments and applications of the split GFP assay is provided by Pedelacq and Cabantous [9].

For monitoring of protein secretion, the split GFP assay can be a simple alternative to activity measurements. It was successfully used for screening of Sec-dependent signal peptides for secretion of a heterologous cutinase and the *Bacillus subtilis* swollenin EXLX1 in *B. subtilis* [10]. Target proteins in the supernatant with an accessible GFP11-tag were detected by addition of externally produced GFP1-10, subsequent chromophore formation and fluorescence measurement. Comparison to activity measurements showed that the assay is a suitable alternative and especially useful for proteins without an established activity assay or without any enzymatic activity [10]. The split GFP assay is applicable in high-throughput screenings, serves as an alternative to established activity assays and can be used in a quantitative manner by combination with activity measurements [4].

Production of GFP1-10 for such applications is mostly done in *E. coli* BL21(DE3) in inclusion bodies from which GFP1-10 can be easily purified and refolded. Protocols for GFP1-10 production in shake flasks and purification have already been published [3, 4]. However, such protocols based on shake flask cultivation are limited in terms of achievable quantity of GFP1-10 and may provide product with varying quality due to limited process control. For high-throughput screening approaches, sufficient quantities of GFP1-10 detector solution is needed. In this contribution, we have developed a fed-batch cultivation process for the GFP1-10 production in laboratory-scale bioreactors.

After purification of GFP1-10 we demonstrate the application of the detector solution to determine heterologous secretion of *Fusarium solani* f. sp. *pisi* cutinase with *Corynebacterium glutamicum*. This cutinase is a well-known hydrolytic enzyme and is used as a model protein to demonstrate applicability of the improved laboratory scale production process of GFP1-10 detector protein. The cutinase gene sequence is modified by adding the 11th $\beta$-sheet of superfolder GFP to the C-terminus (cutinase-GFP11) to enable application of split GFP assay. Secretion of cutinase-GFP11 is enabled by two *B. subtilis* Sec signal peptides with high and low secretion performance of cutinase in *C. glutamicum*. Moreover, aspects of GFP1-10 detector protein stability, storage and assay incubation conditions have been investigated.

## Results and discussion

### Scaling from flask to laboratory-scale bioreactor

Inclusion body-based production of GFP1-10 with *E. coli* BL21(DE3) pET22b-sfGFP1-10 was done in 50 ml lysogeny broth (LB) in shake flasks and in 1 l LB in stirred tank reactors as batch processes. Besides LB, DeLisa defined medium [11] was tested, since it provides improved process control in the bioreactor at lower cost and was successfully used for GFP production in inclusion bodies with *E. coli* as host [12].

**Fig. 1:**
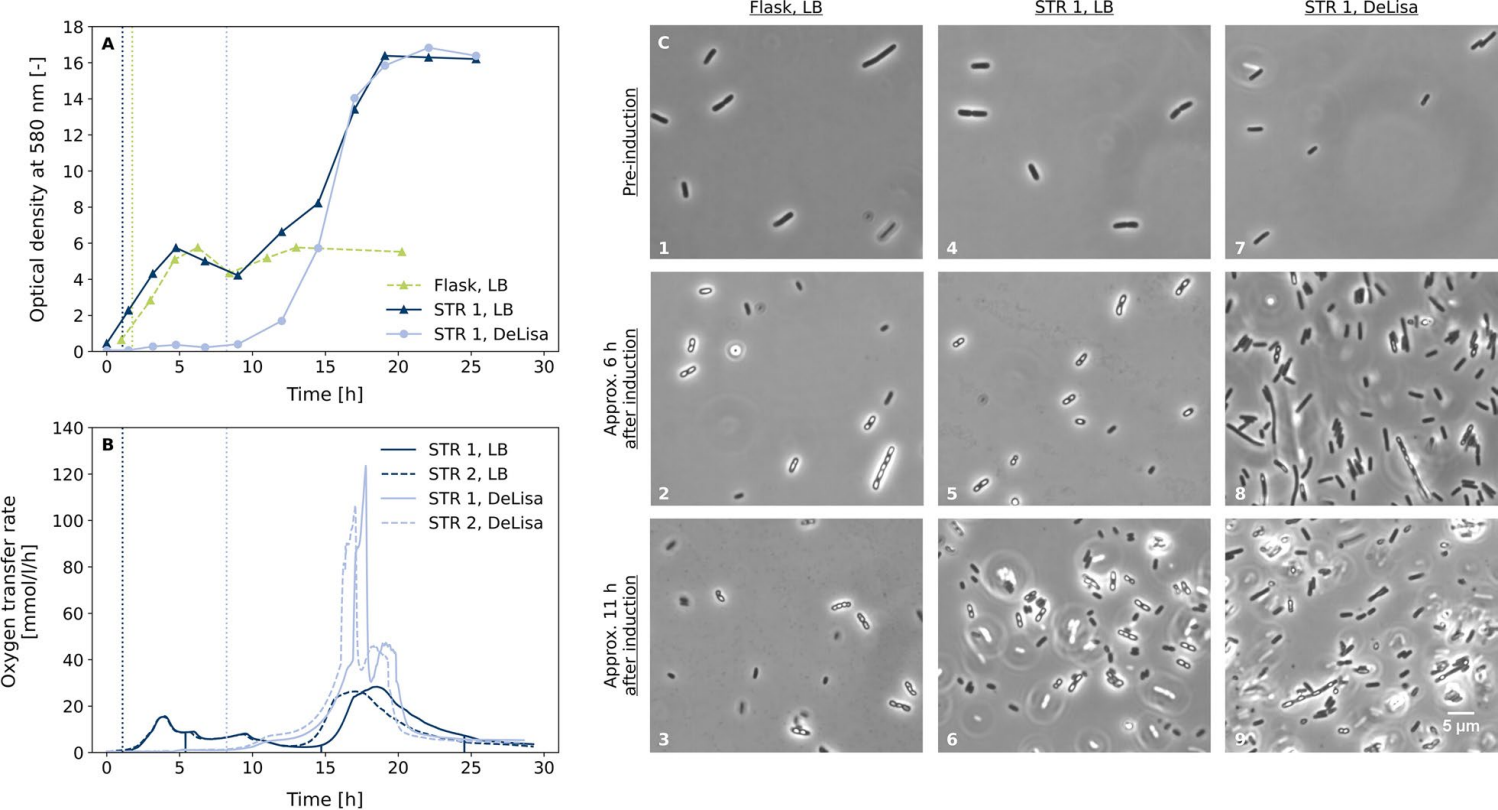
Batch production of GFP1-10 in shake flasks and laboratory-scale bioreactors. **A** Optical density at 580 nm during cultivation of *E. coli* BL21(DE3) pET22b-sfGFP1-10 in shake flask or stirred tank reactor (STR) with LB or DeLisa medium. Dotted lines indicate induction with 200 $\upmu$M IPTG. **B** Oxygen transfer rate during batch fermentation from two biological replicates in LB and DeLisa medium. Dotted lines indicate induction of biological replicates with 200 $\upmu$M IPTG. **C** Microscopic images taken during cultivation at different time points from shake flask with LB (1–3) and bioreactors with LB (4–6) or with DeLisa medium (7–9). Inclusion bodies are the bright spots mainly located at the cell poles. Due to the good comparability of the biological replicates from bioreactors and for a better overview, only the optical densities and microscope images of STR 1 are shown

**Fig. 2:**
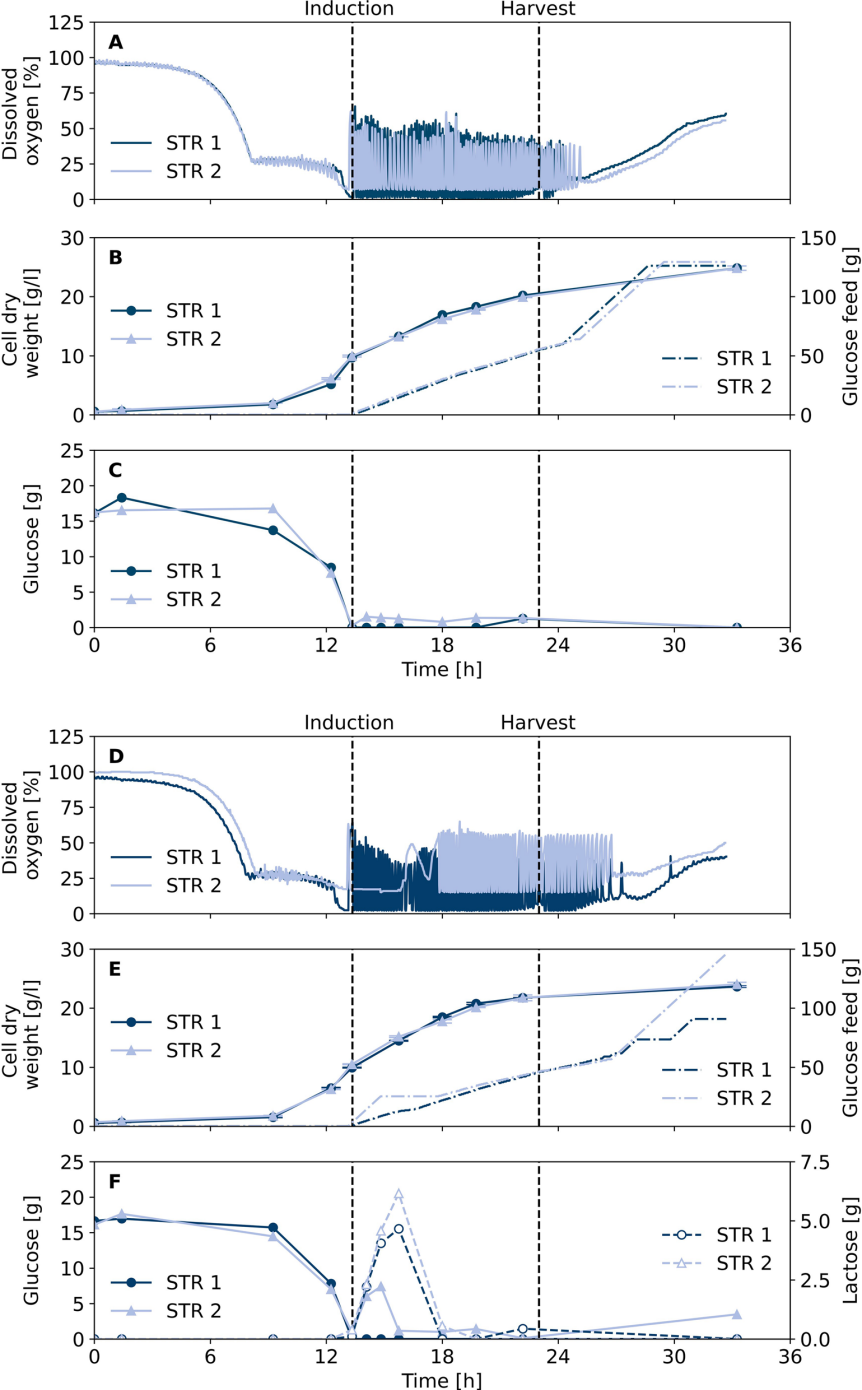
Fed-batch fermentation for production of GFP1-10. On-off settings for constant glucose feeding were triggered by dissolved oxygen signal. GFP1-10 expression in *E. coli* BL21(DE3) pET22b-sfGFP1-10 was induced by constant feeding of IPTG (**A**–**C**) or lactose (**D**–**F**) starting in parallel with glucose feeding until calculated inducer concentrations of 1 mM IPGT or 10 g/l lactose were reached in the bioreactor. Dissolved oxygen and glucose feed were measured for two biological replicates during fed-batch fermentation, respectively. Samples were taken for cell dry weight measurement and HPLC analysis of sugars. Cell samples were harvested after 23 h for purification of GFP1-10 detector protein

In LB shake flask culture low optical densities (OD) of approx. 6 were reached, while OD in batch bioreactor cultures with LB and DeLisa medium was more than twice as high with approx. 17 (Fig. 1A). In LB medium growth started immediately, while there is a lag-phase of about 8 h for the DeLisa defined medium which is followed by exponential growth until OD 17. Although both media exhibit different growth rates, final OD for both media is reached after process time of 18 h. The oxygen transfer rate was calculated from off-gas analytics for the two biological replicates in LB and DeLisa medium (Figure 1B) showing good reproducibility between biological replicates with only a slight time offset. Maximum oxygen transfer rates with DeLisa medium were above 100 mmol/l/h and thus significantly higher than with LB medium which is a consequence of faster growth in this phase. Inclusion body formation was monitored by microscopy (Fig. 1C). In samples before induction, no inclusion bodies were visible under the microscope (Figs. 1C, 1, 4, 7). For cultivations with LB medium more than 50% of the cells seem to contain one or more inclusion bodies 6 h after induction with IPTG. With DeLisa medium, slightly less cells containing inclusion bodies are visible. After 11 h, the number of cells with inclusion bodies dropped, indicating that the harvest time might be a relevant factor.

In terms of batch process time and final biomass concentration, there was no difference between LB and DeLisa medium. Thus, it was decided to select DeLisa medium for the fed-batch process development although slightly reduced inclusion body formation, since it showed higher growth rate with better process control in the bioreactor and reduced media cost.

### Fed-batch process for GFP1-10 production

After the batch process was successfully transferred from shake flasks to bioreactors, glucose feeding was tested to further increase cell densities with DeLisa defined medium. Fed-batch strategy was based on a triggered glucose feed control using the online signal of dissolved oxygen (DO) with on-off setting for the glucose feed pump. Hence, glucose feed pump was activated with a constant feed rate if increased DO indicates substrate depletion and stops if the DO is below a threshold level. With the first start of the glucose feed, the inducer was also constantly fed into the reactor until final inducer concentrations of 1 mM IPTG or 10 g/l lactose were reached. Both inducers were compared for induction of GFP1-10 expression (Fig. 2).

After an initial batch phase the raise of DO above 60% indicated total consumption of batch glucose after 13.5 h and the fed-batch phase was initiated by starting glucose feed control. The added glucose is consumed by the bacteria which increases the oxygen consumption and decreases the DO content, until the glucose feeding is stopped below 10% and started again above 35% DO. This leads to a characteristic fluctuation pattern of DO (Fig. 2A, D). Analysis of supernatant samples showed glucose concentrations close to or at limiting conditions throughout the fed-batch phase (Fig. 2C, F), leading to a final cell dry weight around 25 g/l independent of the induction with IPTG or lactose for each of the two biological replicate cultivations (Fig. 2B, E). In case of lactose as an inducer, the analysis of supernatant samples shows a lactose increase during the first 3.5 h after start of induction in the fed-batch phase followed by rapid decrease until depletion after approx. 4 h for both biological replicates (Fig. 2F). Although the cell dry weight growth profile of both replicates with lactose induction are almost identical, the DO shows a difference. For STR 2 a slight overfeeding in the first 2.5 h of the fed-batch is observed resulting in an intermediary glucose accumulation of approx. 7 g/l. After 13.5 h of cultivation the DO triggered feed was initiated. However, the DO concentration decreased to 14–16% only, so that the glucose feed remained active, which resulted to non-limiting glucose levels during the first 2.5 h. This effect was not present for the STR 1 cultivation and could have resulted from a deviation of the DO sensor signal. Consequently, characteristic oscillating DO profile due to intermittent glucose dosing was observed after glucose and lactose depletion approx. 4 h after fed-batch start.

**Fig. 3:**
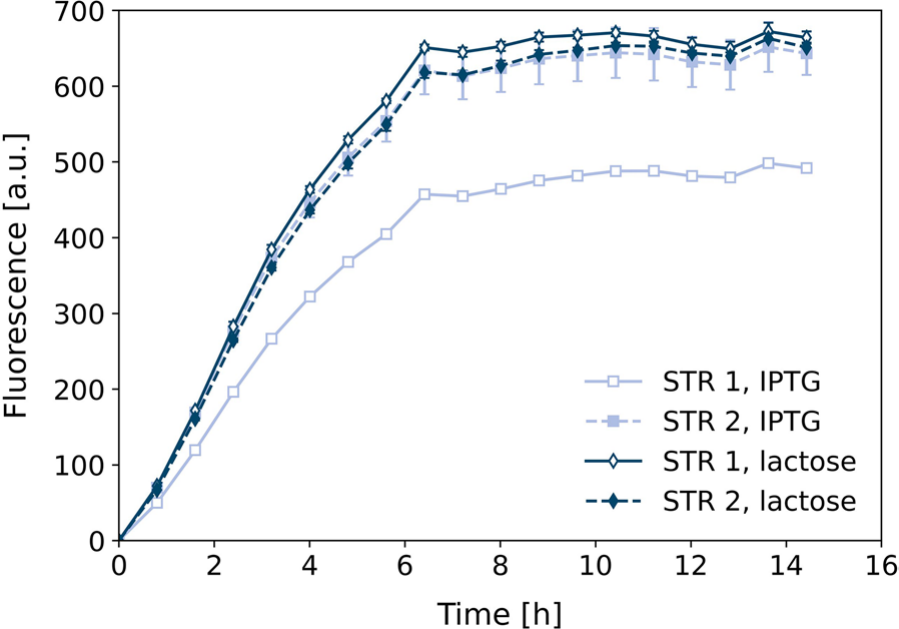
Split GFP assay with GFP1-10 from fed-batch fermentation. Cell samples were taken after 23 h of fed-batch fermentation with IPTG or lactose as inducer. Optical densities were adjusted to 20 and cells were disrupted by French press. GFP1-10 was purified from the inclusion body fraction and used for detection of cutinase-GFP11 in *C. glutamicum* pPBEx2-NprE-Cutinase-GFP11 supernatant by split GFP assay at 20 $^\circ$C

**Fig. 4:**
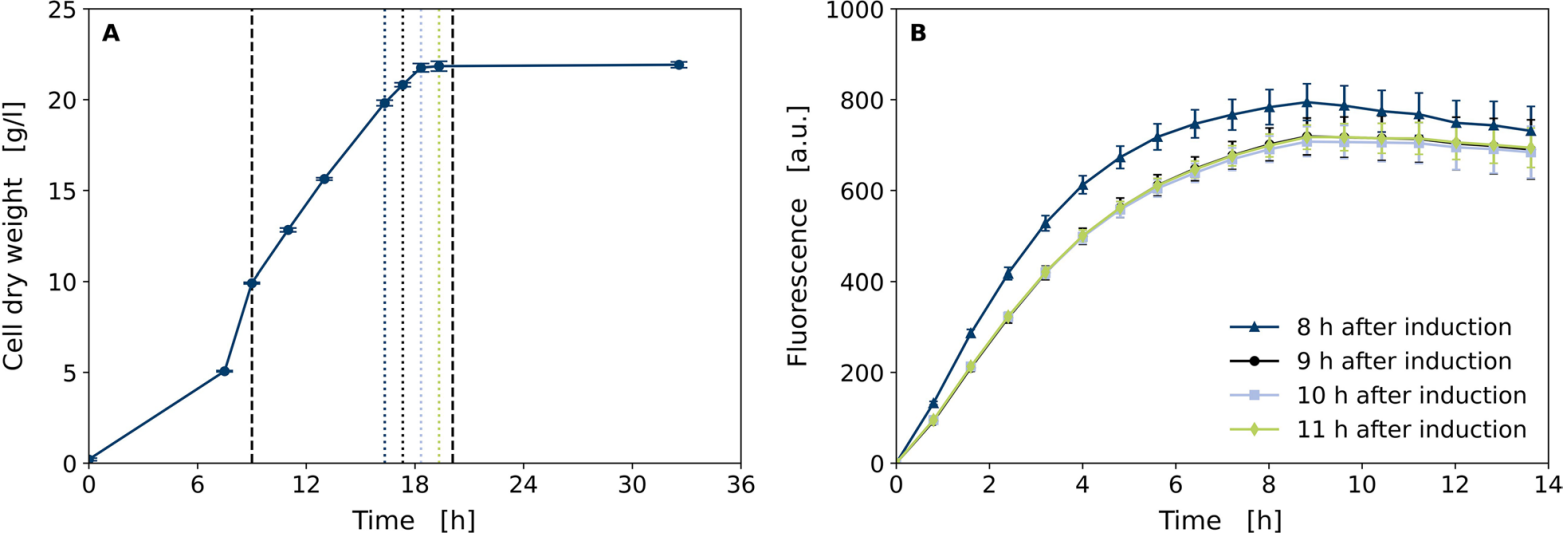
Impact of harvest time in fed-batch GFP1-10 production on quality of GFP1-10 detector solution. Samples for preparation of GFP1-10 detector solution were taken 8, 9, 10 and 11 h after induction of GFP1-10 expression in *E. coli* BL21(DE3) pET22b-sfGFP1-10 with IPTG. **A** Cell dry weight of fed-batch fermentation in DeLisa defined medium. Dashed black lines indicate start and end of feed-phase and dotted lines indicate sampling times. **B** Split GFP assay with detector solutions derived from fermentation samples 8, 9, 10 and 11 h after induction. Supernatant containing cutinase-GFP11 was mixed with the detector solutions, respectively. Data is shown as mean of eight technical replicates with standard deviation

Cells from all cultivations were disrupted by French press and GFP1-10 was purified from the inclusion body fractions following the preparation protocol. Refolded GFP1-10 was applied for split GFP assay with a reference supernatant containing cutinase-GFP11 from *C. glutamicum* pPBEx2-NprE-Cutinase-GFP11 (Fig. 3). Saturation in fluorescence intensity of approx. 650 a.u. was reached after 6.5 h for detector protein solutions from both reactors induced with lactose as well as from STR 2 induced with IPTG. Strikingly, the GFP1-10 detector protein response from STR 1 induced with IPTG showed slower fluorescence increase and lower maximum fluorescence intensity of about 500 a.u. after 13.5 h. Since cultivation data of the biological duplicates induced with IPTG are very similar, the difference in quality of the detector protein solution could have also originated from the multi-step purification protocol.

To conclude, the developed fed-batch process with DO triggered glucose feeding strategy is a suitable way to increase biomass concentration in order to increase total detector protein formation. Moreover, both induction variants, either by IPTG or lactose, led to satisfactory results in terms of detector protein response in the split GFP assay and could be used for fed-batch production processes. From one fed-batch bioreactor cultivation a total amount of final GFP1-10 detector solution can be obtained sufficient to handle up to 385 microtiter plate (MTP, 96-well) screenings.

Since total amount of inclusion body formation and detector protein quality could be dependent on the harvest time point of the cultivation, this was investigated in a fed-batch bioreactor cultivation with IPTG induction (Fig. 4). Samples for GFP1-10 purification were taken 8, 9, 10 and 11 h after the feed start of the inducer IPTG. The process and cell dry weight data were very comparable to the previous experiment (Fig. 2B) indicating good reproducibility of the fed-batch process (Fig. 4A). At all sampling times for GFP1-10 purification, microscopic images show inclusion body formation in the cells (data not shown). GFP1-10 was purified from the inclusion body fraction and used for split GFP assay with supernatant containing cutinase-GFP11 which was obtained from secretory production using *C. glutamicum* pPBEx2-NprE-Cutinase-GFP11 (Figure 4B). Fluorescence signal profiles were very similar for all harvest time points, except for the detector solution derived 8 h after induction. Here, the maximum fluorescence intensity was about 10% higher. Generally, the harvest time seems to show no critical influence in the overall process and all harvesting times tested are suitable for purification of GFP1-10 from the inclusion body fraction. Since total biomass concentration is the highest after 10 h, this latest harvesting time point is prefered. It is very likely, that fed-batch phase could be prolonged in order to achieve even higher detector protein yield, but this is not covered by experimental data so far.

**Fig. 5:**
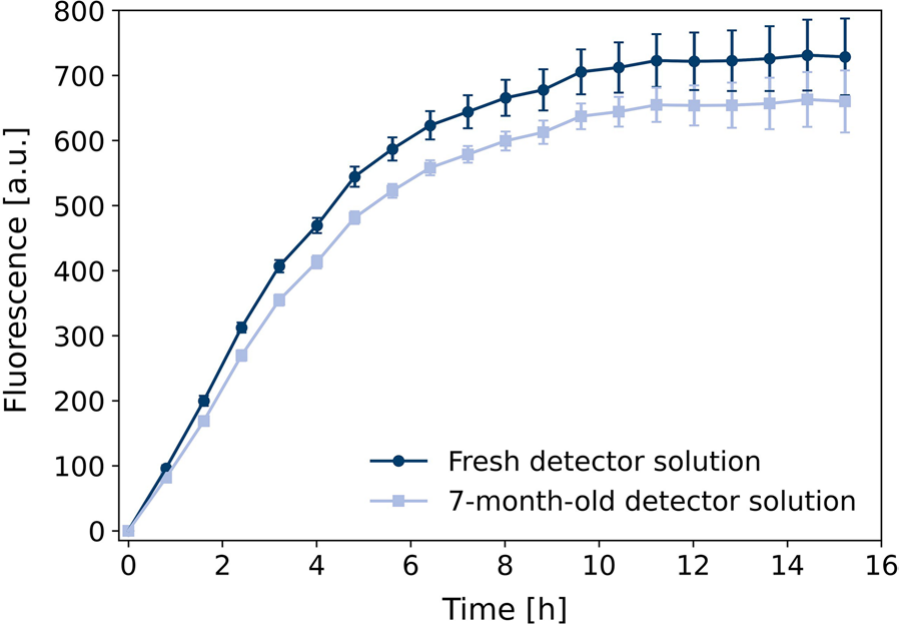
Split GFP assay with stored compared to freshly prepared detector solution. GFP1-10 detector solution was stored at − 20 $^\circ$C for 7 months. For comparison, another detector solution was freshly produced in shake flasks and purified. Both were used for split GFP assay with *C. glutamicum* pPBEx2-NprE-Cutinase-GFP11 supernatant containing cutinase-GFP11

**Fig. 6:**
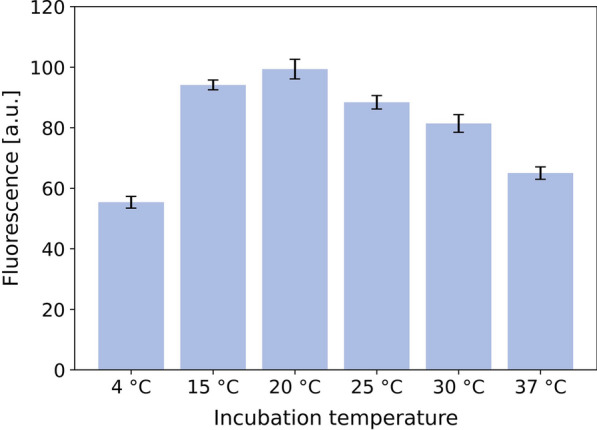
Impact of incubation temperature on split GFP assay. Fluorescence was measured after 16 h of incubation at different temperatures without shaking. Bars indicate mean value of eight replicates with standard deviation. Identical detector solution was mixed with supernatant from *C. glutamicum* pPBEx2-NprE-Cutinase-GFP11 cultivation in shake flask with 6 h of cutinase-GFP11 expression

**Fig. 7:**
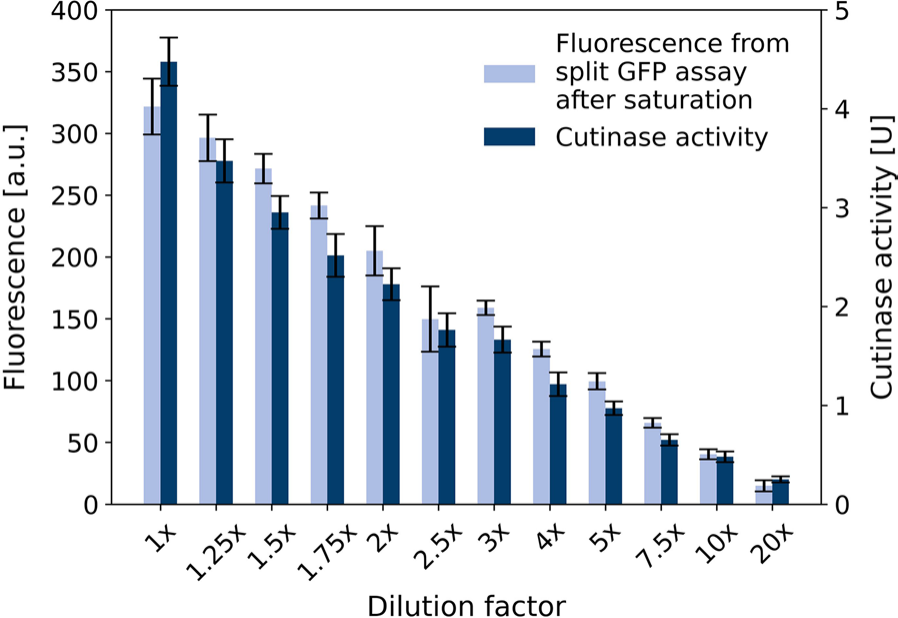
Correlation of split GFP assay and cutinase-GFP11 activity. *C. glutamicum* pPBEx2-NprE-Cutinase-GFP11 supernatant containing cutinase-GFP11 was diluted with different factors and the target protein was detected by split GFP and cutinase activity assay. Error bars for both assays deviated from 8 technical replicates. Fluorescence signal of split GFP assay was measured after saturation of the fluorescence signal after about 10.5 h incubation

**Fig. 8:**
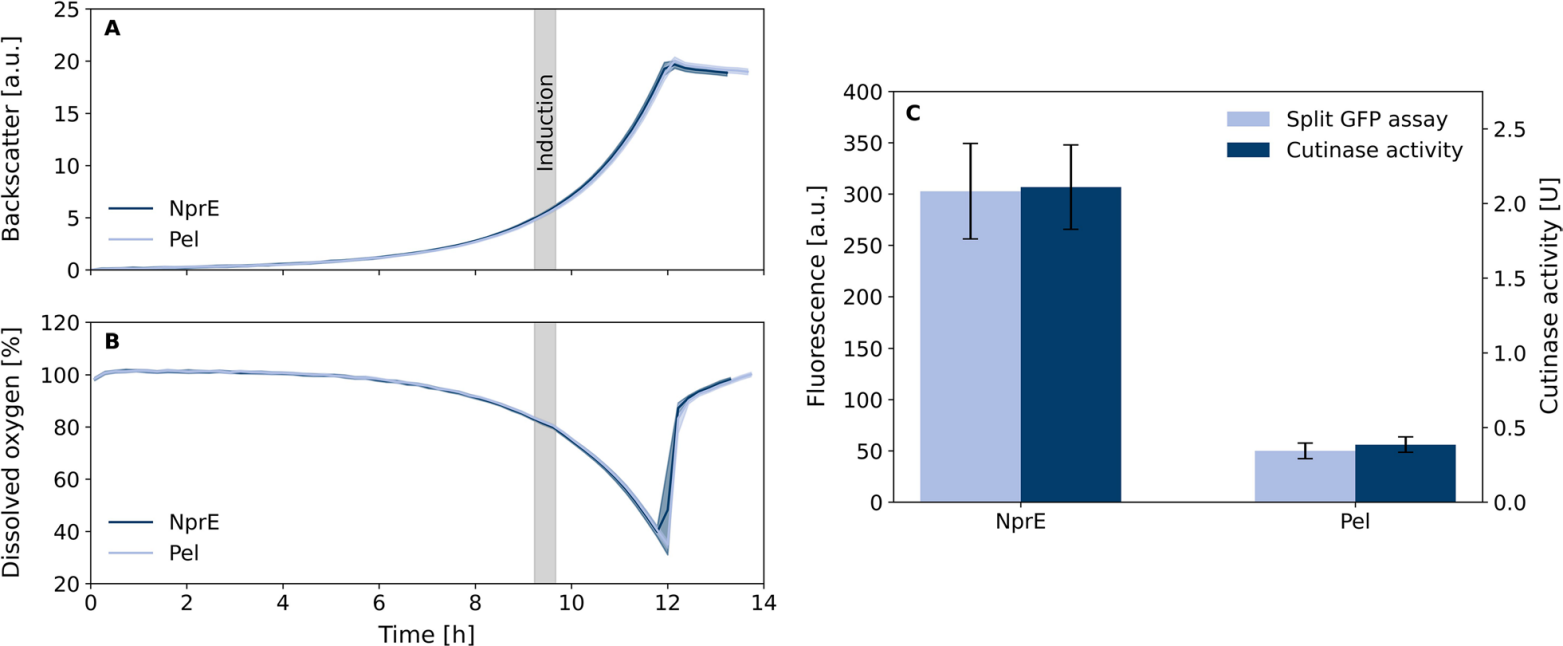
Application of split GFP assay in screening of cutinase-GFP11 secretion. *C. glutamicum* pPBEx2-NprE-Cutinase-GFP11 and pPBEx2-Pel-Cutinase-GFP11 growth curves by backscattered light (**A**) and dissolved oxygen (**B**). Confidence tubes deviated from 24 biological replicates per strain. Each replicate was induced with IPTG individually triggered by backscatter signal. The period in which all samples were induced is highlighted in gray. Cells were harvested 4 h after induction and cutinase-GFP11 in supernatant was detected by split GFP and cutinase activity assay (**C**). Error bars deviated from 24 biological and two technical replicates. Fluorescence signal of split GFP assay was measured after 16 h incubation at 20 $^\circ$C

### Storage stability of GFP1-10

By scaling production of GFP1-10 from shake flask batch to laboratory-scale bioreactor fed-batch process, substantial amounts of detector solution could be obtained from a single bioreactor run sufficient for split GFP assays in approx. 385 MTPs (96-well) for high-throughput screenings. To test the shelf-life of purified GFP1-10 detector solution, refolded GFP1-10 was stored at − 20 $^\circ$C for 7 months before application in the split GFP assay. This is compared to freshly produced and purified detector solution for detection of cutinase-GFP11 in supernatant of *C. glutamicum* pPBEx2-NprE-Cutinase-GFP11 (Fig. 5).

Strikingly, even after 7 months of storage at − 20 $^\circ$C, the GFP1-10 detector solution resulted in fluorescence signals only slightly lower (approx. 10%) than with freshly prepared detector solution. This enables production of a larger stock of detector protein solution with subsequent storage at  − 20$^\circ$C until use for at least 7 months. It is likely that this period could be extended, but this is not covered by the results obtained so far. In a typical screening application, a set of samples from the same screening run are directly compared, making variances in the detector quality over time negligible. Nevertheless, potential differences in the performance of the detector protein solution in terms of the absolute maximum fluorescence signal of the split GFP assay can be compensated. This could be done by correlation of GFP signal with data from activity assay in form of a calibration function to deduce absolute quantitative information.

### Characterization of split GFP assay

The split GFP assay can be used for screening of secreted proteins with *C. glutamicum*. For this, effects of incubation conditions as well as potential influences of supernatant composition must be characterized.

#### Incubation conditions

The split GFP assay was performed with culture supernatant of the cutinase-GFP11 secretion strain *C. glutamicum* pPBEx2-NprE-Cutinase-GFP11 and GFP1-10 detector solution in MTPs without shaking for 16 h at different temperatures with eight replicates each (Fig. 6). The highest fluorescence signals were measured at 20 $^\circ$C while incubation at 4 $^\circ$C resulted in almost half of the fluorescence. It can be speculated that lower temperature hampered proper assembly of the 11th $\beta$-sheet to form the GFP chromophore and that maximum fluorescence seems not be reached after 16 h. Besides, folding and stability of the target protein could also have an impact on the maturation of the chromophore. Temperatures higher than 20 $^\circ$C also show signal decrease. Nevertheless, for further experiments an assay temperature of 25 $^\circ$C was chosen, which is slightly above typical laboratory temperature. This shall avoid conflicts with incubation devices that do not have cooling options.

#### Impact of supernatant composition

Assay robustness against variations in the composition of *C. glutamicum* supernatant was tested. The pH of *C. glutamicum* pPBEx2-NprE-Cutinase-GFP11 supernatant after cutinase-GFP11 secretion was changed from 7.5 to 7.1–7.8 by adding 10 M HCl or 8 M NaOH before split GFP assay. Moreover, the impact of additional 0–250 mM succinate, lactate, glutamate, ketoglutarate and acetate was investigated. Such compounds comprise typical by-product metabolites in microbial cultivations. Neither for the change of pH value, nor the addition of the metabolites a negative impact on the development of GFP split assay fluorescense signal was observed (see Additional file 1: Figures S1 and S2).

#### Correlation of split GFP and activity assay

To ensure that the split GFP assay is a reliable alternative to enzyme activity measurements, *C. glutamicum* pPBEx2-NprE-Cutinase-GFP11 supernatant containing cutinase-GFP11 was used to generate a dilution series which was measured by both, split GFP assay and cutinase activity assay (Fig. 7) . Data show very good comparability between the two assays. This supports the findings with *B. subtilis* as host for secretion, where the split GFP assay was also proven to be a good alternative to activity measurements for the detection of homologous and heterologous target proteins [13].

### Application in screening

To demonstrate the applicability of the improved fed-batch production process for generation and application of detector protein, two *C. glutamicum* strains secreting cutinase-GFP11 with *B. subtilis* signal peptides NprE or Pel were used. *C. glutamicum* pPBEx2-NprE-Cutinase-GFP11 and pPBEx2-Pel-Cutinase-GFP11 were cultivated with 24 biological replicates in a BioLector^®^ Pro microscale cultivation device with backscatter-triggered induction of cutinase-GFP11 expression. The amount of secreted cutinase-GFP11 in supernatant samples was determined by split GFP assay and cutinase activity assay (Fig. 8). All replicate cultivations of both strains showed very similar growth profiles and cutinase-GFP11 expression was induced by IPTG at the same time in the mid exponential phase. However, with respect to the achieved cutinase activity in the supernatant both strains showed very different cutinase-GFP11 secretion performance. While the strain with NprE signal peptide showed much higher split GFP assay response and measured cutinase activity in the range of 300 a.u. and 2.1 U, the strain harboring Pel signal peptide showed much lower values in the range of 50 a.u. and 0.4 U, respectively. The large performance difference was expected and has been confirmed for similar *C. glutamicum* strains for secretory cutinase formation with NprE and Pel signal peptides [14]. With respect to the comparison between activity measurement and split GFP assay, the values were highly comparable in terms of the absolute values as well as the standard error. This gives rise to the conclusion that the developed fed-batch process for GFP1-10 detector protein production is well suited to produce a larger stock of detector protein solution, which can be stored up to 7 months with minor loss of fluorescense response in the range of 10% only.

## Conclusions

The production of detector protein GFP1-10 could be successfully scaled from shake flask batch to laboratory-scale bioreactor fed-batch process. By fed-batch fermentation with intermittent glucose feed triggered by DO, detector solution for up to 385 MTPs (96-well) screenings could be obtained. GFP1-10 detector solution could be stored at − 20 $^\circ$C for at least 7 month with very little performance loss.

Applicability of split GFP assay in high-throughput secretion screening of cutinase-GFP11 with *C. glutamicum* as host was verified. The split GFP assay can be easily automated as no appropriate sample dilution is needed and only the detector solution needs to be provided.

In addition, the split GFP assay offers excellent opportunities for data normalization to reliably compare secretion performance within a screening round or after correlation with enzyme activity data measured for absolute calibration as this is demonstrated in the correlation of split GFP fluorescense versus cutinase in the application study. The biggest advantage of the split GFP assay is that it can be easily adapted to other target proteins. As long as the GFP11-tag is accessible, nothing needs to be changed in the screening workflow. Even proteins without enzymatic activity or without an established activity assay can be detected without elaborate alternatives like ELISA assays.

**Table 1:** Plasmids used in this study

Name	Description	Resource
pET22b-sfGFP1-10	pET22b(+) with *sfgfp1-10* gene under control of P_T7_	[13]
pPBEx2-NprE-Cutinase-GFP11	*F. solani* f. sp. * pisi cutinase* gene with N-terminal *B. subtilis* signal peptide NprE and C-terminal GFP11-tag cloned into pPBEx2 [20] via PstI and SacI, P_tac_	This study
pPBEx2-Pel-Cutinase-GFP11	*F. solani* f. sp. * pisi cutinase* gene with N-terminal *B. subtilis* signal peptide Pel and C-terminal GFP11-tag cloned into pPBEx2 [20] via PstI and SacI, P_tac_	This study

## Methods

### Strains and media

Plasmids used in this study are shown in Table 1. *E. coli* BL21(DE3) pET22b-sfGFP1-10 [13] was used for expression of detector protein GFP1-10. Cultivations were either carried out in LB with Miller’s modifications [15] or in DeLisa defined medium [11], both supplemented with 100 $\upmu$g/ml ampicillin. Plasmids pPBEx2-NprE-Cutinase-GFP11 and pPBEx2-Pel-Cutinase-GFP11 for cutinase-GFP11 secretion with *B. subtilis* Sec-specific signal peptides NprE and Pel were kindly provided by Dr. Patrick Backes (Forschungszentrum Jülich GmbH, Germany). Sequences of these constructs are available in Additional File 1.

*C. glutamicum* 13032 was transformed by electroporation as previously described [16]. Brain Heart Infusion (Carl Roth, Karlsruhe, Germany) or CGXII [17] with 20 g/l glucose was used for cultivation each containing 30 $\upmu$g/ml kanamycin for plasmid stability.

### Offline analysis

Optical density was measured at 580 nm (*E. coli*) or at 600 nm (*C. glutamicum*) in a UV-1800 spectrophotometer (Shimadzu, Japan) with 0.9% (w/v) NaCl as blank and for appropriate dilution of samples. For cell dry weight analysis, pre-weighed reaction tubes were filled with 2 ml of sample and centrifuged at 21500 $\times$g and 4 $^\circ$C for 10 min. The cell pellet was dried at 90 $^\circ$C for 24 h and stored in a desiccator before determination of cell dry weight. Samples from GFP1-10 production were analyzed by microscopy (Eclipse Ti2, Nikon, Japan) with 100$\times$ oil immersion objective to detect inclusion body formation. Offline pH was measured with an electrode calibrated by two points (pH 4 and pH 7).

Glucose and lactose concentration in fermentation samples were measured by HPLC (HP 1100, Agilent technologies, USA) with an organic acid resin (Metab-AAC, 300 $\times$ 8 mm, Isera, Germany) at 25 $^\circ$C. Elution was carried out with 0.1 M sulfuric acid at a flow rate of 0.6 ml/min. 20 $\upmu$l of sample or standard were injected and sugars were detected with a refractometer. Cultivation supernatant was stored at − 20 $^\circ$C until use, 10-fold diluted and sterile filtered. Standard solutions of glucose and lactose ranging from 0.1–20 g/l were freshly prepared.

### Production of GFP1-10

Two precultures were incubated at 37 $^\circ$C and 250 rpm with 25 mm shaking diameter in baffled flasks with 10-fold volume compared to the filling volume. 10 ml LB were inoculated with a single colony from agar plate. The second preculture was either 100 ml LB or DeLisa depending on the medium of the main culture. For batch experiments, the first preculture was incubated for 4 h and 1 ml was used to inoculate a second preculture which was incubated for another 16 h. For fed-batch experiments, the first preculture was incubated for about 10 h and 300 $\upmu$l were used to inoculate a second preculture which was then incubated for 6 h.

Main cultures were inoculated to an OD of 0.05. They were either carried out in 500 ml baffled flasks filled with 50 ml LB under incubation conditions of the preculture or in 1.5 l DASGIP^®^ bioreactors (Eppendorf, Germany) with two Rushton-type impellers (6 blades, 1 cm height, 3 cm distance). Bioreactors were equipped with DASGIP^®^ modules TC4SC4 for temperature and agitation control, PH4PO4 for control of DO and pH, MF4 for mass flow controlled gassing, MP8 for control of feed flow rates and GA4 exhaust analyzer (all by Eppendorf, Germany). DO was measured with VisiFerm DO 225 optodes (Hamilton, Switzerland) and pH with 405-DPAS-SC-K8S electrodes (Mettler Toledo, USA).

In batch-mode, 1 l LB or DeLisa medium was incubated at 37 $^\circ$C with an initial agitation speed of n_0_ = 400 rpm and air flow rate of q_in, 0_ = 0.1 vvm. The pH was set to 6.7 and controlled with 1 M sodium hydroxide and 1 M hydrochloric acid. DO was kept $\ge$ 30% by varying the agitation speed n = 400–1200 rpm and air flow rate q_in_ = 0.1–2 vvm. GFP1-10 expression was induced by adding 200 $\upmu$M IPTG.

Fed-batch experiments comprised a batch phase before glucose feeding and induction triggered by DO. This batch phase was conducted in the same way as batch fermentation, but with an initial volume of 800 ml DeLisa medium with 20 g/l glucose. Phosphoric acid (30%, v/v) and ammonium hydroxide were used for pH control. Constant feedings of 500 g/l glucose and inducer solution (10 mM IPTG or 100 g/l lactose) were started at the end of batch phase (DO $\ge$ 60%) with 30 ml/h pump rate. Feeding of inducer solution was stopped once a final concentration of 1 mM IPTG or 10 g/l lactose were reached in the bioreactor, respectively. The glucose pump was operated under on-off control with limits set to 10–35% DO. Sterile Antifoam 204 (Sigma-Aldrich, USA) was added if necessary.

### Cell disruption and GFP1-10 purification

Cell pellet was resuspended in buffer consisting of 100 mM Tris-HCl pH 7.4, 100 mM NaCl and 10% (v/v) glycerol (TNG buffer) to an OD of 20. Cell disruption was carried out with French press (15000 psi, four runs), followed by centrifugation (4000 $\times$g, 4 $^\circ$C, 10 min). Pellet containing GFP1-10 was purified three times by resuspension in TNG buffer and subsequent centrifugation. As described by [4], the resulting pellet containing inclusion bodies was resuspended in 1 ml urea each 75 mg pellet and centrifuged (4000 $\times$g, 4 $^\circ$C, 20 min). 400 $\upmu$l supernatant each were mixed with 10 ml TNG buffer for refolding of GFP1-10 and stored at − 20 $^\circ$C until use. Additional 10 ml of TNG buffer were added directly before split GFP assay to get the final detector solution.

### Cutinase-GFP11 secretion

10 ml BHI in a 100 ml baffled flask was inoculated with 1 ml cryoconserved *C. glutamicum* pPBEx2-NprE-Cutinase-GFP11 or pPBEx2-Pel-Cutinase-GFP11 and incubated for 6 h at 30 $^\circ$C, 250 rpm and 25 mm shaking diameter. A second preculture with 10 ml CGXII with 10% (v/v) BHI medium in a 100 ml baffled flask was inoculated with 100 $\upmu$l of the first preculture and incubated for 16 h at the same conditions.

The main culture was carried out in a BioLector^®^ Pro (m2p-labs, Germany) at 30 $^\circ$C, 1400 rpm and $\ge$ 85% relative humidity. 800 $\upmu$l CGXII were inoculated to an OD of 0.2 in a FlowerPlate^®^ with optodes (m2p-labs, Germany). Cutinase-GFP11 expression in each well was induced individually with 100 $\upmu$M IPTG at a backscatter value corresponding to 4 g/l cell dry weight. Cells were harvested after 4 h (2898 $\times$g, 4 $^\circ$C, 6 min) and supernatant was stored until cultivation of all wells was finished.

Alternatively, 50 ml CGXII in a 500 ml baffled flask were inoculated with 300 $\upmu$l of the second preculture. 100 $\upmu$M IPTG were added at an OD of 0.3–0.4. Cells were harvested after 6 h of cutinase-GFP11 expression for 10 min at 4000 $\times$g and 4 $^\circ$C. Supernatant was stored at − 20 $^\circ$C until use. If cutinase-GFP11 expression lasted for 16 h, some changes were made. The first preculture was inoculated with 50 $\upmu$l cryoconserved *C. glutamicum*, incubated for 16 h and the second preculture for 6 h. The main culture was inoculated with 500 $\upmu$l of the second preculture. Cutinase-GFP11 expression was done in shake flasks for 16 h unless stated otherwise.

### Split GFP assay

As previously described, 180 $\upmu$l of detector solution were mixed with 20 $\upmu$l of cutinase-GFP11 containing supernatant in a black MTP with clear bottom [13]. Self-assembly of GFP was recorded over a time period of at least 13 h in an MTP reader (Infinite^®^ M Nano, Tecan, Switzerland) by measurements at an excitation wavelength of $\lambda$ = 485 nm and an emission wavelength of $\lambda$ = 535 nm. Between measurements, plate was shaken inside the MTP reader (linear mode, 887 rpm) at 25 $^\circ$C unless stated otherwise.

### Cutinase activity assay

Activity of cutinase-GFP11 was determined spectrophotometrically as described elsewhere [18] by degradation of 4-nitrophenyl palmitate (4NPP) as substrate analog [19]. Briefly, 9 parts of reaction buffer (2.3 g/l Na-desoxycholate, 1.1 g/l gum arabic in 55 mM potassium phoshate buffer, pH 8) were mixed with 1 part 3 g/l 4NPP in isopropanol. 200 $\upmu$l of this reaction mix were filled into wells of a MTP and pre-warmed to 37 $^\circ$C. Supernatant was diluted 500-fold with 55 mM potassium phosphate buffer (pH 8) and 40 $\upmu$l were pipetted into two wells filled with the reaction mix for technical duplicates. Formation of 4-nitrophenol (4NP) was measured at 410 nm and 37 $^\circ$C over 40 min in an MTP reader (Infinite^®^ M Nano, Tecan, Switzerland). Triplicates of 40 $\upmu$l 4NP in a concentration range of 0–2 mM were mixed with 200 $\upmu$l reaction mix and absorption was measured to convert absorption into product concentration.

## Supplementary Information

**Additional file 1:Figure S1.** Impact of supernatant pH on split GFP assay. **Figure S2.** Impact of different metabolites in *C. glutamicum* supernatant on split GFP assay. **Table S1.** Gene sequences of cutinase-GFP11 with *B. subtilis* signal peptides NprE and Pel.

## Data Availability

All data generated or analyzed during this study are included in this article and its Additional file 1.
