# Supplementary material for: Scaling production of GFP1-10 detector protein in E. coli for secretion screening by split GFP assay
Source: Microb Cell Fact. 2021 Sep 30;20:191. doi: 10.1186/s12934-021-01672-6 (PMC8482599; doi:10.1186/s12934-021-01672-6)
Supplement: Supplementary file 1 — Additional file 1:Figure S1. Impact of supernatant pH on split GFP assay. Figure S2. Impact of different metabolites in C. glutamicum supernatant on split GFP assay. Table S1. Gene sequences of cutinase-GFP11 with B. subtilis signal peptides NprE and Pel. [file 12934_2021_1672_MOESM1_ESM.pdf]

Additional File 1

Supplementary Figures

Müller et al. 2021

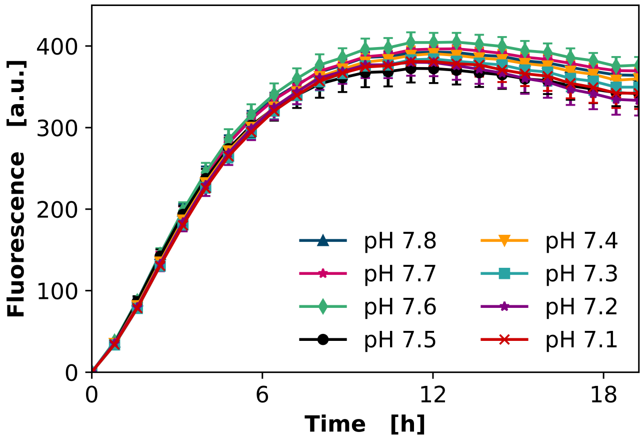

Figure S1: Impact of supernatant pH on split GFP assay. Original pH of *C. glutamicum* pPBEx2-NprE-Cutinase-GFP11 supernatant containing cutinase-GFP11 was 7.5. Acid (10 M HCl) or base (8 M NaOH) was added to change pH of supernatant to 7.1–7.8 before split GFP assay. Data is shown as mean of three technical replicates with standard deviation.

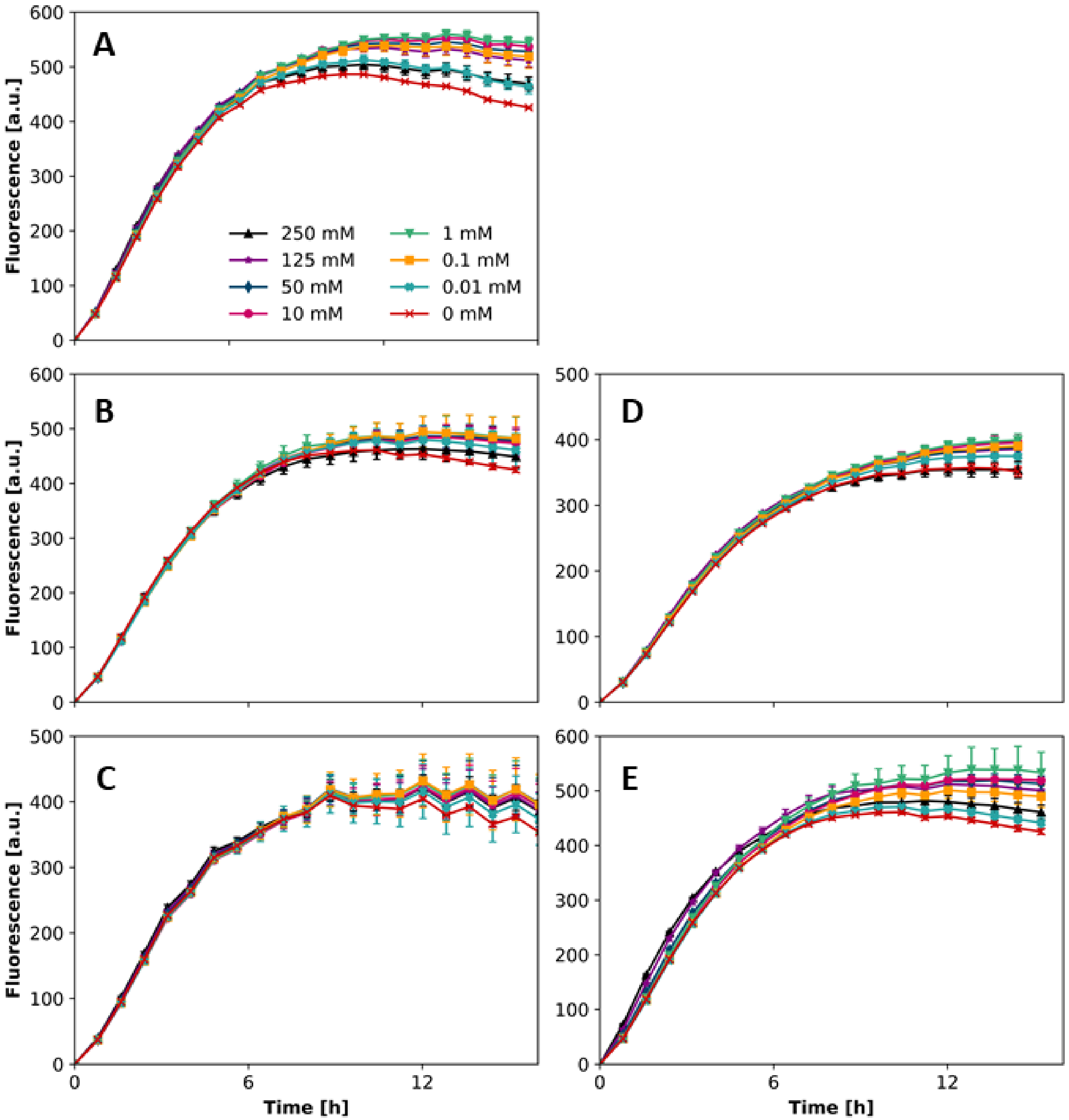

Figure S2: Impact of different metabolites in *C. glutamicum* supernatant on split GFP assay. Succinate (A), lactate (B), glutamate (C), ketoglutarate (D) and acetate (E) were added with final concentrations of 250–0.1 mM to *C. glutamicum* pPBEx2-NprE-Cutinase-GFP11 supernatant containing cutinase-GFP11. Data is shown as mean of three technical replicates with standard deviation.

Table S1: Gene sequences of cutinase-GFP11 with *B. subtilis* signal peptides NprE and Pel.

Gene	DNA sequence (5' $\rightarrow$ 3')
NprE-Cutinase-GFP11	ATGGGTTTAGGTAAGAAATTGTCTGTTGCTGTCGCTGCTTCGTT<br>TATGAGTTTATCAATCAGCCTGCCAGGTGTTCAGGCTGCTGAAT<br>TCGCGCCTACTAGTAACCCTGCTCAGGAGCTTGAGGCGCGCCA<br>GCTTGGTAGAACAACTCGCGACGATCTGATCAACGGCAATAGC<br>GCTTCCTGCGCCGATGTCATCTTCATTTATGCCCGGGGTTCAAC<br>AGAGACGGGCAACTTGGGAACTCTCGGTCCTAGCATTGCCTCC<br>AACCTTGAGTCCGCCTTCGGCAAGGACGGTGTCTGGATTCAGG<br>GCGTTGGCGGTGCCTACCGAGCCACTCTTGGAGACAATGCTCT<br>CCCTCGCGGTACCTCTAGCGCCGCAATCAGGGAGATGCTTGGT<br>CTCTTCCAGCAGGCCAACACCAAGTGCCCCGACGCGACTTTGA<br>TCGCCGGTGGCTACAGCCAGGGTGCTGCACTTGCGGCCGCCT<br>CCATCGAGGACCTCGACTCGGCCATTCGTGACAAGATCGCCGG<br>AACTGTTCTGTTCGGCTACACCAAGAACCTACAGAACCGTGGC<br>CGAATCCCCAACTACCCTGCCGACAGGACCAAGGTCTTCTGCA<br>ATACAGGAGATCTCGTTTGTACTGGTAGCTTGATCGTTGCTGCA<br>CCTCACTTGGCTTATGGTCCTGATGCTCGGGGCCCTGCCCCTG<br>AGTTCCTCATCGAGAAGGTTCGGGCTGTCCGTGGTTCTGCTCTT<br>ATCGGCTCTGATGGCGGCTCTGGCGGCGGCTCTACATCTCGTG<br>ATCACATGGTTCTTCATGAATACGTTAACGCTGCTGGCATCACA<br>TAA
Pel-Cutinase-GFP11	ATGAAAAAAGTGATGTTAGCTACGGCTTTGTTTTTAGGATTGAC<br>TCCAGCTGGCGCGAACGCAGCTGAATTCGCGCCTACTAGTAAC<br>CCTGCTCAGGAGCTTGAGGCGCGCCAGCTTGGTAGAACAACTC<br>GCGACGATCTGATCAACGGCAATAGCGCTTCCTGCGCCGATGT<br>CATCTTCATTTATGCCCGGGGTTCAACAGAGACGGGCAACTTG<br>GGAACTCTCGGTCCTAGCATTGCCTCCAACCTTGAGTCCGCCTT<br>CGGCAAGGACGGTGTCTGGATTCAGGGCGTTGGCGGTGCCTA<br>CCGAGCCACTCTTGGAGACAATGCTCTCCCTCGCGGTACCTCT<br>AGCGCCGCAATCAGGGAGATGCTTGGTCTCTTCCAGCAGGCCA<br>ACACCAAGTGCCCCGACGCGACTTTGATCGCCGGTGGCTACAG<br>CCAGGGTGCTGCACTTGCGGCCGCCTCCATCGAGGACCTCGAC<br>TCGGCCATTCGTGACAAGATCGCCGGAACTGTTCTGTTCGGCT<br>ACACCAAGAACCTACAGAACCGTGGCCGAATCCCCAACTACCC<br>TGCCGACAGGACCAAGGTCTTCTGCAATACAGGAGATCTCGTT<br>TGTACTGGTAGCTTGATCGTTGCTGCACCTCACTTGGCTTATGG<br>TCCTGATGCTCGGGGCCCTGCCCCTGAGTTCCTCATCGAGAAG<br>GTTCGGGCTGTCCGTGGTTCTGCTCTTATCGGCTCTGATGGCG<br>GCTCTGGCGGCGGCTCTACATCTCGTGATCACATGGTTCTTCAT<br>GAATACGTTAACGCTGCTGGCATCACATAA
